# Editorial: Glycan diversity in fungi, bacteria, and sea organisms

**DOI:** 10.3389/fcimb.2015.00044

**Published:** 2015-05-19

**Authors:** Eliana Barreto-Bergter

**Affiliations:** Laboratório de Química Biológica de Microrganismos, Departamento de Microbiologia Geral, Instituto de Microbiologia, Universidade Federal do Rio de JaneiroRio de Janeiro, Brasil

**Keywords:** fungal glyconjugates, marine invertebrates, glycosaminoglycans, lipopolysaccharide, biological activity

The cell surface of fungi, bacteria, and sea organisms is highly glycosylated. These glycans are oligo- or polysaccharide molecules that can be secreted or attached to protein or lipids forming glycoconjugates. They present extraordinary structural diversity that could explain their involvement in many fundamental cellular processes, including growth, differentiation, and morphogenesis. Considerable advances have been made on the structural elucidation of these glycans. Their primary structures were determined based on a combination of mass spectrometry and NMR spectroscopy techniques. The combination of these sensitive and powerful techniques has allowed us to increase our structural knowledge of a wide variety of glycans expressed by different fungi, bacteria and sea organisms.

The Research topic “Glycan diversity in fungi, bacteria, and sea organisms” covered important aspects related to polysaccharides, glycoproteins and glycolipids from different organisms and their biological functions.

Some contributions to this Research topic have highlighted the importance of glycosaminoglycans analogs with unique structures from different marine invertebrates. Many sulfated fucans (SFs), sulfated galactans (SGs), and glycosaminoglycans (GAGs) of new structures have been characterized and described (Vieira and Mourão, 1988; Mourão, 2004; Pomin and Mourão, 2008). Pomin and Mourão (2014) have made clear the relevance of certain structural combinations of sulfation and glycosylation to the anticoagulant activity of the marine carbohydrates of well-defined chemical structures.

Pomin (2014) has described the most important marine carbohydrates with therapeutic actions, as well as their main structural and medical properties.

Pavão (2014) emphasized that whereas these marine organisms will be a source of new heparin analogs with significant therapeutic effect in thrombosis, inflammation and cancer in the future will depend on the economic pressure of the pharmaceutical industry and the increasing demand for new natural drugs with less undesired side effects to treat specific diseases.

Plouguerné et al. (2014) reviewed the literature on the glycolipids from seaweeds and their potential biotechnological applications. The most reported biological activities for glycolipids from seaweeds were antibacterial, antitumor, and antiviral activities, enhancing the pharmacological potential of these compounds. Antifouling, and antiherbivory activities were already reported for glycolipids from *Sargassum muticum* and *Fucus vesiculosus*, respectively (Deal et al., 2003; Plouguerné et al., 2010).

The diversity of the composition of the fungal cell surface and important aspects related to structure and function of fungal glycans has also been reviewed in this Research topic. These contributions highlighted the importance of surface molecules of fungal cells for the fungal pathogenesis, physiology, and immune recognition.

Guimarães et al. (2014) focused on glycan structures carried on sphingolipids of pathogenic/opportunistic fungi, and aspects of their biological significance have been discussed.

A review from Barreto-Bergter and Figueiredo (2014) showed that the variety of carbohydrate structures present in the different fungal pathogens offers exceptional targets for the innate immune recognition which has evolved to recognize specific fungal glycans through a plethora of different receptors.

Burjack et al. (2014) demonstrated a structural diversity of the polysacharides from *Fonsecae monophora* isolated from clinical and environmental origins.

Mannoproteins with different molecular masses were identified and characterized as *Cryptococcus neoformans* immunoreactive antigens by Teixeira et al. (2014) with potential cryptococcosis vaccine candidates.

In addition to glycans from sea organisms and fungal cells, this Research topic included a mini review on the lipopolysaccharide from bacteria. Serrato (2014) has described the structure of diazotrophic bacteria and highlights the importance of these glycolipids in the microbe-plant interaction.

All these articles strongly indicate that knowledge on structure and functions of glycans from fungi, bacteria and sea organisms may open new perpectives allowing to identify specific targets for new generation of antifungal drugs, development of new classes of immunomodulators, antigens, and adjuvants and also marine carbohydrate-based drug development.

## Conflict of interest statement

The author declares that the research was conducted in the absence of any commercial or financial relationships that could be construed as a potential conflict of interest.
